# Intravenous immunoglobulin in the treatment of scleromyxedema associated with monoclonal gammopathy^؆^

**DOI:** 10.1016/j.abd.2025.501149

**Published:** 2025-07-08

**Authors:** Amanda Cochlar Medeiros Perrella, Alexandre Michalany, Mário Cezar Pires, Cassio Oliveira Lima

**Affiliations:** a Service of Dermatology, Complexo Hospitalar Padre Bento de Guarulhos, Guarulhos SP, Brazil; b Department of Dermatopatology, Laboratório Paulista de Dermatopatologia, Jardim Paulista, São Paulo, SP, Brazil

*Dear Editor,*

Scleromyxedema is a chronic disease of the spectrum of primary cutaneous mucinosis that affects young adults of both sexes.^1^ According to Rongioletti et al.,^2^, ^3^ the diagnostic criteria are: generalized papular and sclerodermiform rash, mucin deposition in the upper dermis, fibroblast proliferation and collagen fiber thickening, monoclonal gammopathy (90% monoclonal gammopathy of undetermined significance [MGUS]) and normal thyroid function.

Extracutaneous involvement may occur, with neurological, rheumatological, cardiovascular, gastrointestinal, respiratory, renal and ocular alterations. “Dermatoneuro syndrome” is a severe complication characterized by fever, epileptic seizures and coma. The mortality rate of scleromyxedema reaches over 20%.^1^

The pathogenesis and action mechanism of human intravenous immunoglobulin (IVIG) in this disorder is still a matter of debate. It has been observed that serum from affected patients induces fibroblast proliferation *in vitro* and that, after IVIG infusion, there is a reduction in IL-17 levels and TGF-β gene expression.^4^, ^5^

The present report describes a 34-year-old female patient who developed a disseminated rash with waxy, pruritic papules, some assuming a linear arrangement, on thickened, difficult-to-fold skin (Fig. 1). She showed infiltration of the face and ears, longitudinal furrows in the glabella and madarosis. Histopathology showed fibroblast proliferation, collagen fiber thickening, and mucin deposits in the dermis with colloidal iron staining (Fig. 2).

**Fig. 1:**
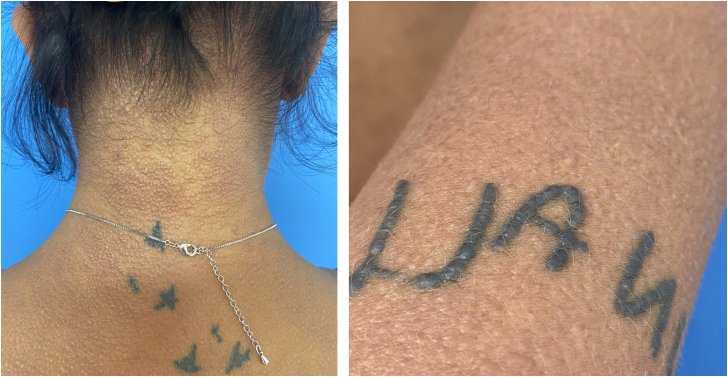
Linear arrangement of papules.

**Fig. 2:**
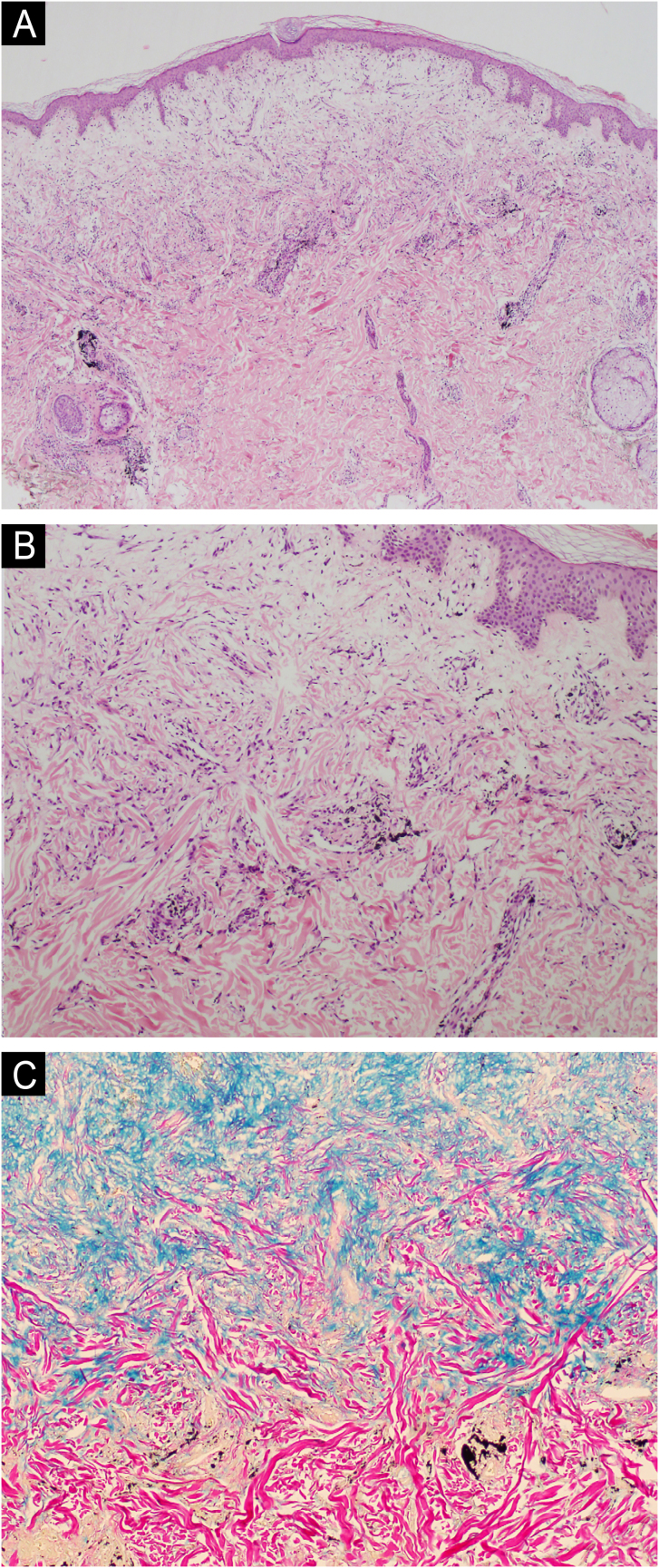
(A) Pallor of the superficial dermis (Hematoxylin & eosin, ×40); (B) Increased number of fibroblasts and collagen fiber thickening in the middle dermis (Hematoxylin & eosin, ×100); (C) Mucin deposition (Colloidal iron, ×100).

Based on the physical and pathological examination, the investigation was started for monoclonal gammopathies with electrophoresis and immunofixation of serum and urinary proteins, which revealed a monoclonal peak in gamma globulin (IgG) and IgG kappa monoclonality; myelogram, which showed 8% of plasma cells; bone marrow (BM) immunophenotyping, showing 0.8% of plasma cells with kappa light-chain monoclonality; in addition to BM karyotype, the ratio between serum free light chains, measurement of serum immunoglobulins, thyroid function, renal function, calcium metabolism, blood count and bone inventory by tomography, all without alterations. These complementary exams led to the diagnosis of MGUS, an asymptomatic premalignant condition that eventually transforms into Multiple Myeloma (MM).

Six infusions of IVIG 2 g/kg were performed, each lasting three days and with an interval of four to six weeks, with significant improvement in the condition (Fig. 3, Fig. 4). This result was similar to the report by Guarneri et al. with eight patients and an 81.6% improvement in the modified Rodnan score.^6^

**Fig. 3:**
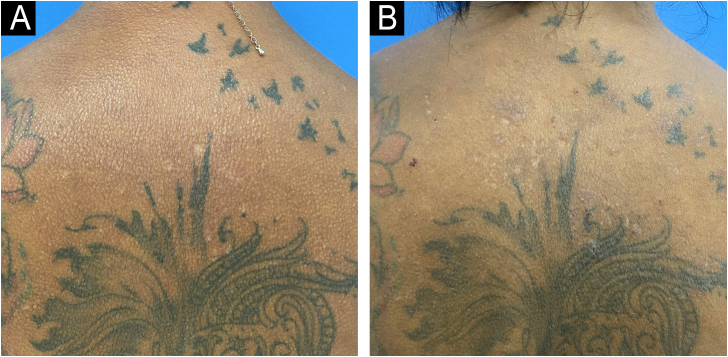
Infiltration improvement and papule reduction on the upper back (A) before treatment and (B) after treatment.

**Fig. 4:**
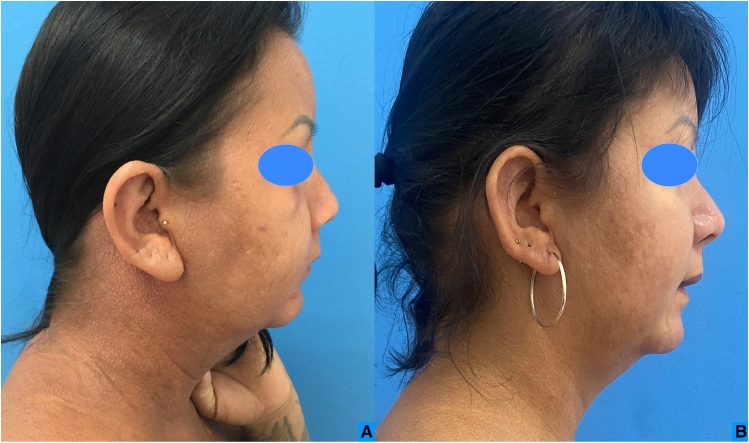
Infiltration improvement of the face in profile (A) before treatment and (B) after treatment.

The patient will continue to receive IVIG indefinitely since most patients experience recurrence if treatment is interrupted. There is only one case report in which there was spontaneous remission.^1^, ^7^, ^8^

Since this is a rare disease, randomized controlled clinical trials are not feasible. Based on systematic reviews and two prospective uncontrolled clinical trials, the best scientific evidence available regarding treatment advocates in favor of high-dose IVIG every four to six weeks. Thalidomide with corticosteroids can be used as a second-line treatment. Bortezomib and, more drastically, autologous bone marrow transplantation (BMT) are third-line treatments. Melphalan, despite being mentioned, has a high risk of hematological malignancy development with increased mortality due to the treatment itself.^1^, ^6^, ^9^

Despite the association with monoclonal gammopathies, a review with 17 patients demonstrated sustained remission of scleromyxedema in only 2% of patients after BMT, even though the hematological disorder had been cured.^8^

Bortezomib is a reversible inhibitor of the 26S proteasome pathway that prevents the activation of the NF-κB transcription factor and induces apoptosis in neoplastic cells. Its main indication is for the treatment of MM. Like IVIG, the action mechanism in scleromyxedema is unknown. Despite having been the therapy with the longest remission period described in the literature to date, emerging as a new treatment perspective, for now, it is limited as a third-line recommendation due to the few published studies.^1^, ^9^, ^10^

Although rare, scleromyxedema has its importance due to its chronicity and high morbidity and mortality. Its low prevalence limits the establishment of research protocols for the development of new therapies with better cost-benefit. Although the strengthening of genetic and immunological knowledge facilitates this endeavor, case reports, such as this one, demonstrating the success of the employed therapy, provide data to the literature for future systematic reviews.

## Authors' contributions

Amanda Cochlar Medeiros Perrella: Design and planning of the study; collection of data, or analysis and interpretation of data; drafting and editing of the manuscript; or critical review of important intellectual content; collection, analysis, and interpretation of data; Intellectual participation in the propaedeutic and/or therapeutic conduct of the studied cases; critical review of the literature; approval of the final version of the manuscript.

Alexandre Michalany: Collection of data, or analysis and interpretation of data; effective participation in research orientation; approval of the final version of the manuscript.

Mário Cezar Pires: Design and planning of the study; effective participation in research orientation; intellectual participation in the propaedeutic and/or therapeutic conduct of the studied cases; approval of the final version of the manuscript.

Cassio Oliveira Lima: Design and planning of the study; collection of data, or analysis and interpretation of data; drafting and editing of the manuscript; or critical review of important intellectual content; collection, analysis, and interpretation of data.

## Financial support

None declared.

## Conflicts of interest

None declared.
